# The Hidden Impact of the COVID-19 Pandemic on Routine Childhood Immunization Coverage in Cameroon

**DOI:** 10.3390/vaccines11030645

**Published:** 2023-03-14

**Authors:** Yauba Saidu, Pietro Di Mattei, Sangwe Clovis Nchinjoh, Nnang Nadege Edwige, Bernard Nsah, Nkwain Jude Muteh, Shalom Tchokfe Ndoula, Rakiya Abdullahi, Chen Stein Zamir, Andreas Ateke Njoh, Amani Adidja, Sidy Ndiaye, Owens Wiwa, Emanuele Montomoli, Sue Ann Costa Clemens

**Affiliations:** 1 Clinton Health Access Initiative Inc., Yaounde P.O. Box 2664, Cameroon; 2 Institute for Global Health, University of Siena, 53100 Siena, Italy; 3 Gavi, The Vaccine Alliance, 1202 Geneva, Switzerland; 4 Faculty of Medicine, The Hebrew University-Hadassah Braun School of Public Health and Community Medicine, Jerusalem 91120, Israel; 5 Expanded Program on Immunization, Cameroon Ministry of Public Health, Yaounde P.O. Box 2084, Cameroon; 6 Global Vaccine Team, Clinton Health Access Initiative Inc., Boston, MA 02127, USA; 7 School of Global Health and Bioethics, Euclid University, Bangui BP 157, Central African Republic; 8 Faculty of Medicine and Biomedical Sciences, University of Yaoundé 1, Yaounde B.P. 337, Cameroon; 9 Regional Office for Africa, World Health Organization, Brazzaville P.O. Box 06, Congo; 10 Department Molecular Medicine, University of Siena, Via Aldo Moro 3, 53100 Siena, Italy; 11 VisMederi srl, Via Ferrini 53, 53035 Siena, Italy; 12 Department of Pediatrics, University of Oxford, Oxford OX1 2JD, UK

**Keywords:** COVID-19 impact, childhood vaccination, Cameroon

## Abstract

**Background**: The third round of the global pulse survey demonstrated that the abrupt and rapid progression of the COVID-19 pandemic significantly disrupted childhood immunization in many countries. Although Cameroon has reported over 120,000 COVID-19 cases, the reported national childhood vaccination coverage during the pandemic seems to have increased compared to that during the pre-COVID-19 period. Indeed, the first dose of the diphtheria, tetanus, and pertussis-containing vaccine (DTP-1) coverage increased from 85.4% in 2019 to 87.7% in 2020, and DTP-3 coverage increased from 79.5% in 2019 to 81.2% in 2020. The paucity of literature on the impact of COVID-19 on childhood vaccination in COVID-19 hotspot regions poses a challenge in developing a context-specific immunization recovery plan, hence the need to conduct this study. **Methodology**: We conducted a cross-sectional study using 2019 (pre-pandemic period) and 2020 (pandemic period) district childhood immunization data from the DHIS-2 database, weighted using completeness for each data entry against regional data completeness in 2020. Based on COVID-19 incidence, two hotspot regions were selected, with all districts (56/56) included in the final analysis. The Chi-square test was used to compare DTP-1 and DTP-3 coverage during the pre-pandemic and pandemic periods. **Results**: In the two hotspot regions, 8247 children missed DTP-1, and 12,896 children did not receive DTP-3 vaccines in the pandemic period compared to the results from the pre-pandemic period. Indeed, there was a significant drop in DTP-1 and DTP-3 coverage of 0.8% (*p* = 0.0002) and 3.1% (*p* = 0.0003), respectively, in the Littoral Region. Moreover, the Centre Region reported a 5.7% (*p* < 0.0001) and 7.6% (*p* < 0.0001) drop in DTP-1 and DTP-3 coverage, respectively. Most districts in the hotspot regions reported a decline in childhood immunization access (62.5%) and utilization (71.4%). Indeed, in the Littoral Region, 46% (11/24) and 58% (14/24) of districts experienced decreased vaccination access and utilization, respectively. Meanwhile, 75% (24/32) and 81% (26/32) of districts in the Centre Region experienced a drop in vaccination access and utilization, respectively. **Conclusion**: This study reported a situation where the national immunization indicators mask the impact of COVID-19 on childhood immunization in heavily hit regions. Therefore, this study presents valuable information for ensuring continuous vaccination service delivery during public health emergencies. The findings could also contribute to developing an immunization recovery plan and informing policy on future pandemic preparedness and response.

## 1. Background

Complex emergencies and natural disasters are associated with outbreaks of infectious diseases due to disruptions in health service delivery, including vaccination and nutrition [1]. Although there is limited literature on the impact of pandemics on essential health service delivery, the COVID-19 pandemic forced countries to observe social isolation, physical distancing, lockdowns, curfews, and quarantines. This may have posed a population health risk similar to the case of complex emergencies and natural disasters. Since 2019, the SARS-CoV-2 virus has rapidly spread from China, infecting over 650 million people, with 6.6 million deaths recorded globally as of 22 December 2022 [2].

The abrupt and rapid progression of the COVID-19 pandemic has caused significant disruptions in essential health service delivery in many countries, reversing past efforts to improve health indicators [3,4,5]. Indeed, according to the third round of the global pulse survey on the continuity of essential health services during the COVID-19 pandemic, over 90% of countries reported a serious continuous disruption in the delivery of essential health services [6]. Moreover, 53% of countries reported persistent disruptions in primary health care, with about 40% experiencing increased backlogs during the second half of 2021 [6]. These disruptions are mainly due to decreased care seeking in 25% of countries, but also unintended disruptions resulting from the lack of healthcare resources and intentional service delivery modifications in one-third of surveyed countries [6]. Moreover, a systemic review suggested a significant decline in vaccination coverage due to COVID-19, leading to a four-fold increase in polio cases in polio-endemic countries [7]. According to the authors, factors contributing to the observed decline include: fear of being exposed to the virus at healthcare facilities, restriction on city-wide movements, a shortage of workers, and diversion of resources from child health to address the pandemic, among others [7].

Cameroon, with an estimated population size of 27 million in 2022, has recorded over 120,000 COVID-19 cases and about 2000 COVID-19-related deaths, yet the reported national childhood routine vaccination coverage seems to have improved compared to the pre-COVID-19 period [2]. For instance, the first dose of the diphtheria, tetanus, pertussis-containing vaccine (DTP-1) coverage increased by almost 2 percentage points (pp), rising from 85.4% in 2019 to 87.7% in 2020. Similarly, the third dose of DTP-containing vaccine (DPT-3) increased from 79.5% in 2019 to 81.2% in 2020. The observed increase in coverage suggests an increased access to, and utilization of, vaccination services during the COVID-19 pandemic [8]. This observation runs contrary to what has been reported by previous studies, which all showed serious disruptions in regards to other essential health system indicators. These authors reported serious disruptions in blood donation services, utilization of radiology units, geriatric consultations, pediatric hospitalization, and HIV service utilization, among others [9,10,11,12,13,14,15]. Another cross-sectional study assessed the impact of COVID-19 on immunization services in a single hospital setting in Cameroon—posing a problem for result generalizability [16]. In the current study we aimed to contribute by filling the existing knowledge gap concerning the impact of COVID-19 on routine childhood immunizations in Cameroon, which might be critical in ensuring continuous vaccination service delivery during public health emergencies. In addition, findings from this study will provide salient recommendations that could contribute to developing the COVID-19 recovery plan and informing policy on future pandemic preparedness and response.

## 2. Methodology

### 2.1. Study Design and Setting

This cross-sectional study compared childhood routine immunization access and utilization in the pre-pandemic period (2019) to the pandemic period (2020). The study considered aggregated secondary district-level data on routine childhood immunization from the District Health Information System (DHIS)-2. All districts found in the top two COVID-19 hotspot regions were considered for analysis.

### 2.2. Key Operational Definitions

In this study, childhood routine immunization access was measured using diphtheria, pertussis, tetanus first dose (DTP-1) vaccination coverage as an indicator, while routine immunization utilization was based on DTP-3 vaccination coverage, DTP-1 and DTP-3 were used as indicators to align with the Cameroon ministry of public health’s definitions for vaccination access and utilization; thus allowing for result dissemination and use by policy makers. Moreover, we defined COVID-19 hotspot regions as those with the highest number of cumulative COVID-19 cases as of July 2022.

### 2.3. Sampling and Data Collection

Based on administrative data, the Littoral Region, with 32,995 COVID-19 cases representing 28.27% of national cumulative cases, and the Centre Region, with 36,506 COVID-19 cases representing 31.28% of national cumulative cases, were considered COVID-19 hotspot regions, and were included in the study. The secondary data on annual district DTP-1 and DTP-3 coverages in all districts in the two hotspot regions were extracted from DHIS-2 and prepared for analysis. A total of 56 of 189 health districts (29.6%) in Cameroon were included in the study, notably 24 from the Littoral Region and 32 from the Centre Region.

### 2.4. Data Management and Analysis

The data were exported as a Microsoft Excel 2013 worksheet into R Statistical Software (v4.1.2; R Core Team 2021) for statistical analysis. District vaccination coverage was weighted using completeness of the data entry according to regional data completeness in 2020. Data on regional and district data entry completeness reported in the DHIS-2 database (in percentages) was downloaded and cleaned, and the formulas below were used to calculate the adjusted vaccine coverages.

(1) $$Adjusted\;DTP-1\;coverage=\frac{Regional\;data\;completeness}{District\;data\;completeness}\;\times \;DTP-1\;coverage$$

(2) $$Adjusted\;DTP-3\;coverage=\frac{Regional\;data\;completeness}{District\;data\;completeness}\;\times \;DTP-3\;coverage$$

Using the Chi-square test, we compared the adjusted DTP-1 and DTP-3 coverages in 2019 (pre-pandemic) and 2020 (pandemic). The 2020 data were considered the observed outcome, and the 2019 data reflected the expected outcome in the analysis. A *p*-value < 0.05 was considered statistically significant.

### 2.5. Ethical Considerations

This study did not involve any individual-level data, so ethical clearance was not required.

## 3. Results

This study included annual DTP-1 and DTP-3 vaccination coverages from all districts in the COVID-19 hotspot regions, representing one-third of districts (56/189) in Cameroon. Based on the DHIS-2 data quality assessment tool, data completeness in the pre-pandemic period was 96% and 100% in the Littoral and Centre regions, respectively. In addition, in the pandemic period, data exhibited 91% and 94% completeness in the Littoral and Centre regions, respectively.

**Figure 1:**
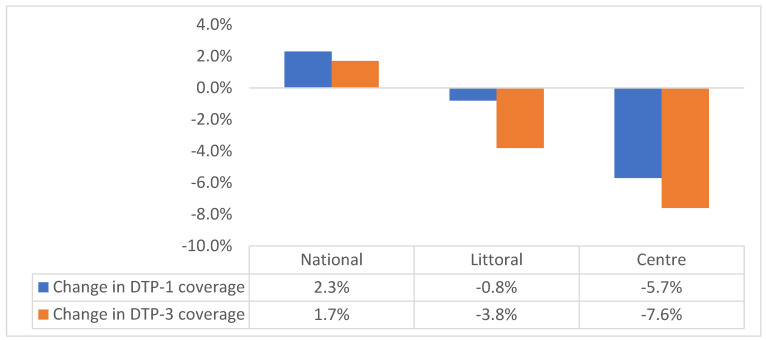
Change in DTP-1 and DTP-3 coverage in hotspot regions during the pandemic.

**Figure 2:**
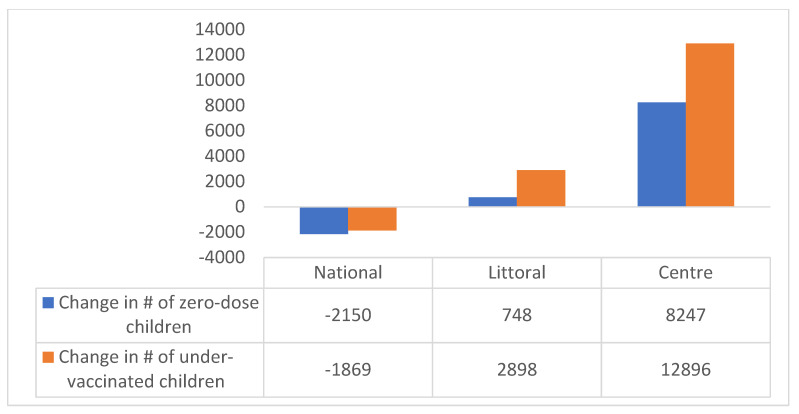
Number of children who missed vaccination in hotspot regions and contrasting national gain in children vaccinated during the pandemic.

Basing on these assumptions, our results unveiled a significant drop in vaccination coverage of 3.3% and 5.4% in DTP-1 and DTP-3 coverages, respectively. As a result, 8247 children missed DTP-1, and 12,896 children did not receive DTP-3 vaccines in the pandemic period compared to the results for pre-pandemic period in the two hotspot regions. This is contrary to the trend in national data, as shown in Figure 1 and Figure 2. Additionally, most districts reported a decline in childhood immunization access (62.5%) and utilization (71.4%), ranging from a drop of 0.1% to 43.7% in vaccination coverage.

As presented in Table 1 and Table 2, in the Littoral Region, 46% (11/24) of districts experienced a decreased vaccination access, which ranged from 2.6% to 29.4%. Moreover, 58% (14/24) of districts experienced decreased vaccine utilization, which ranged from 0.1% to 28.3%. The adjusted drop in vaccination coverage in the Littoral Region was 0.8% (*p* = 0.0002) and 3.1% (*p* = 0.0003) for DTP-1 and DTP-3 vaccination coverages, respectively. This implies that about 748 children missed DTP-1 vaccines, and 2898 children the Littoral Region did not receive DTP-3 vaccines during the pandemic compared to the pre-pandemic period.

Meanwhile, as shown in Table 3 and Table 4, the Centre Region experienced a 5.7% (*p* < 0.0001) drop in DTP-1 and a 7.6% (*p* < 0.0001) drop in DTP-3 coverage, with about 7498 children missing DTP-1 and 9998 others missing DTP-3 during the pandemic compared to the pre-pandemic period. This decrease in DTP-1 vaccination coverage was reported in 75% (24/32) of districts, ranging from a 2.1% to a 31.6% drop in coverage. Likewise, 81% (26/32) of districts showed a decreased DTP-3 vaccination coverage in the pandemic period, with a 2.5% to a 43.7% drop in coverage.

## 4. Discussion

Analyzing data from the two COVID-19 hotspot regions in Cameroon revealed a significant drop in DTP-1 coverage of 3.3% and in DTP-3 coverage of 5.4%. This drop resulted into 8247 and 12,896 children missing out on their DTP-1 and DPT-3 vaccines, respectively, in the pandemic period compared to the pre-pandemic period. Moreover, the DTP series vaccination dropout rate increased from 7.5% to 9.3% in the Centre Region and from 3.7 to 6.0 in the Littoral Region. Our findings run contrary to national administrative data, which suggested improved childhood vaccination access and utilization during the pandemic period. Improvement at the national level is understandable, because vaccination has mainly been driven by the organization of periodic intensification of routine immunization (PIRIs) in many districts of some regions, particularly those in the Southwest and Northwest regions [17]. Moreover, several vaccination campaigns were organized in other regions in response to VPD epidemics, coupled with the introduction of a second dose of measle and rubella vaccine [18,19]. This may have had a significant impact on the coverage of other antigens, increasing national vaccination coverage.

Despite this improvement in national coverage, which was purely driven by the PIRIs, our findings clearly align with those reported by the third round of the global pulse survey on the impact of COVID-19 on immunization services [6]. The survey revealed that 70% (64/91) of participating countries reported disruptions in routine immunization services, with 18% (16/91) experiencing severe disruptions between February and August 2020 [20]. Moreover, a study conducted in a tertiary hospital in Cameroon revealed a decreased demand for childhood immunization services during the COVID-19 pandemic, with a significant drop in the coverage of DTP-containing vaccines [16]. Other studies reported a similar decline in immunization indicators during the pandemic [21,22].

The significant drop in vaccination coverage in our study can be explained by the advent of a novel pandemic that encountered an unprepared and weak health system, hence the grave challenges in meeting the demands of pandemic control. This led to task shifting in favor of the pandemic, creating an unintended negative impact on essential health services, such as routine immunization. Additionally, sub-optimal training of clinicians regarding routine patient care, including vaccination amid the pandemic, created, the fear of contracting COVID-19 when offering health services [9]. This fear was worsened by inadequate personal protective equipment and standards of operation to keep the disease in check in a clinical setting [9]. Delays in COVID-19 confirmatory diagnosis due to limited test kits and diagnostics targeted every patient presenting in a clinical setting with upper or lower respiratory tract symptoms as a suspected case, leading to poor care, even for patients presenting with other ailments [9]. This complexity and uncertainty associated with contracting the COVID-19 infection may have created a spillover effect of COVID-19 stigma and hesitancy toward other routine essential health care services, including immunization services.

Although districts recorded varying degrees of change in vaccination access and the utilization of tracer indicators between the pre-pandemic and pandemic period, more than two-thirds of them reported a drop in DTP-1 (62.5%) and DTP-3 coverages (71.4%). Up to a 31.6% and a 43.7% drop in DTP-1 and DTP-3 coverage, respectively, was reported in some districts. This finding lends support to the results of a cross-sectional study in Senegal that showed a significant decrease in immunization uptake at the health facility level [23]. This further emphasizes the need for a real-time assessment tool to be used at the different tiers of vaccination service delivery, including health facilities. This is important because aggregated data at higher administrative levels may mask prevailing low performance at lower operational levels. The role of such a tool in data-driven decision making at all levels is invaluable.

The region most heavily hit by the COVID-19 pandemic (the Center Region) recorded a higher drop in vaccination access and utilization in the pandemic period, with more districts reporting a drop in vaccination indicators compared to the results from the Littoral Region. The drop ranged from 5.7% (*p* < 0.0001) to 0.8% (*p* = 0.0002) in the Centre and Littoral regions, respectively. There was also a significant drop in the utilization of immunization services in both regions, and estimates stood at 7.6% (*p* < 0.0001) and 3.1% (*p* = 0.0003) in the Center and Littoral regions, respectively. In a country such as Cameroon, with limited health resources, this piece of information may be helpful to prioritize regions and districts with higher decline in RI indicators, as this may guide the development of a post-COVID-19 recovery plan to reverse the impact of the pandemic on key RI indicators. This finding can also be employed in informing policy on future pandemic management.

Despite the potential usefulness and application of our findings, there are certain limitations that must be acknowledged. These limitations are essentially linked to data completeness on the DHIS-2 platform, which was the main source of data for our study. Based on the DHIS-2 data quality assessment tool, data completeness in the pre-pandemic period was 96% and 100% in the Littoral and Centre regions, respectively; however, during the pandemic period, data completeness was at 91% and 94% in the Littoral and Centre regions, respectively. In this study, we adjusted this limitation by weighting the data against regional data completeness. The data weighting may have introduced bias in some districts by disproportionately increasing or decreasing vaccination coverage.

Based on our findings, we will first recommend a further survey in a sample of these districts to identify factors associated with the decline in vaccination coverage during the pandemic. Second, we recommend the development and validation of a digital tool that can support the early detection of the impact of a pandemic on RI variables at all health system tiers. These two recommendations may be valuable in developing tailored strategies to detect and reverse-inverse trends of the pandemic on RI performance.

## 5. Conclusions

This study presented a practical scenario in Cameroon, where national data masked the impact of the COVID-19 pandemic on childhood immunization in COVID-19 hotspot regions. Indeed, the study revealed a remarkable decrease in vaccination access and utilization in the two COVID-19 hotspot regions, with an increase in DTP-series dropout rate during the pandemic compared to the pre-pandemic period. Therefore, there is a resounding need to develop and implement recovery and catch-up immunization strategies to mitigate the impact of the pandemic on routine childhood immunization in these regions. To ensure the continuity of childhood routine immunization in future pandemics, there is need to set up a system that will drive the early detection of the impact of the pandemic on immunization services. Moreover, a digital tool that would evaluate the impact of the pandemic at all operational levels in real-time will be of great value. This will help unveil the pandemic’s true impact and support data-driven decision making. Additionally, the study findings can be leveraged to inform policies on sustaining immunization services during future pandemics in Cameroon.

## Figures and Tables

**Table 1:** DTP-1 coverage variations in the pre-pandemic and pandemic period in the Littoral Region.

District	2019 Weight	2019 Coverage	Adjusted 2019 Coverage	2020 Weight	2020 Coverage	Adjusted 2020 Coverage	Variation *	Adjusted Variation *
Abo	0.98	131.3	128.2	1.12	107.8	120.7	−23.5 *	−7.5 *
Bangue	1.08	88.2	95.0	1.06	91.4	97.3	3.2	2.3
Boko	0.93	49.1	45.8	0.91	47.3	43.2	−1.8 *	−2.6 *
Bonassama	1.02	96	97.5	1.00	101.6	101.5	5.6	4.0
Cite Des Palmiers	1.18	101.3	119.6	1.13	81.6	92.2	−19.7 *	−27.3 *
Deido	0.91	95.7	87.1	0.88	65.3	57.7	−30.4 *	−29.4 *
Dibombari	0.97	101.7	98.5	0.92	135.6	124.9	33.9	26.4
Edea	1.04	76	79.1	1.12	82.5	92.8	6.5	13.7
Japoma	1.16	72.6	84.2	1.14	85.8	97.5	13.2	13.3
Logbaba	1.26	89.8	112.7	1.41	84.5	119.4	−5.3 *	6.7
Loum	0.98	119.5	117.2	0.98	116.5	113.6	−3 *	−3.6 *
Manjo	1.15	86.9	99.8	1.12	74.5	83.4	−12.4 *	−16.5 *
Manoka	2.43	15.4	37.4	1.04	23.8	24.8	8.4	−12.6 *
Mbanga	1.11	72.4	80.2	1.15	63.4	73.1	−9 *	−7.0 *
Melong	0.97	100.4	97.8	1.10	88.8	97.8	−11.6 *	0.0
Ndom	1.07	45.1	48.1	1.39	43.4	60.5	−1.7 *	12.4
New Bell	0.97	70.8	68.4	0.99	94.8	93.9	24	25.5
Ngambe	1.09	51.6	56.4	0.91	65.2	59.3	13.6	3.0
Njombe Penja	1.42	89	126.2	1.13	95.1	107.1	6.1	−19.0 *
Nkondjock	0.99	101.9	101.1	0.95	103.8	98.2	1.9	−2.9 *
vvNkongsamba	0.99	106.6	105.3	1.07	99.9	107.0	−6.7 *	1.6
Nylon	0.91	94.3	86.2	0.93	96.9	90.4	2.6	4.2
Pouma	1.09	100.3	109.6	1.46	80.3	116.9	−20 *	7.3
Yabassi	1.27	76.7	97.6	1.32	65.3	86.4	−11.4 *	−11.2 *

* The negative sign (−) shows a drop in coverage after the pandemic compared to before, while a positive sign (+) indicates an increase in coverage.

**Table 2:** DTP-3 coverage variations in the pre-pandemic and pandemic period in the Littoral Region.

District	2019 Weight	2019 Coverage	Adjusted 2019 Coverage	2020 Weight	2020 Coverage	Adjusted 2020 Coverage	Variation *	Adjusted Variation *
Abo	0.98	122.4	119.5	1.12	108.9	121.9	−13.5 *	2.4
Bangue	1.08	89.3	96.2	1.06	90.4	96.2	1.1	0.0
Boko	0.93	52.3	48.8	0.91	49.6	45.3	−2.7 *	−3.4 *
Bonassama	1.02	92.7	94.1	1.00	96.3	96.2	3.6	2.0
Cite Des Palmiers	1.18	99.8	117.8	1.13	80.1	90.5	−19.7 *	−27.2 *
Deido	0.91	88.6	80.6	0.88	59.2	52.3	−29.4 *	−28.3 *
Dibombari	0.97	94.7	91.7	0.92	133.5	123.0	38.8	31.3
Edea	1.04	72.9	75.9	1.12	71.4	80.3	−1.5 *	4.4
Japoma	1.16	70.8	82.1	1.14	77.1	87.6	6.3	5.5
Logbaba	1.26	82.4	103.4	1.41	71.3	100.8	−11.1 *	−2.7 *
Loum	0.98	109.4	107.3	0.98	109.1	106.4	−0.3 *	−0.9 *
Manjo	1.15	89.2	102.5	1.12	75.9	85.0	−13.3 *	−17.5 *
Manoka	2.43	8.4	20.4	1.04	8.3	8.6	−0.1 *	−11.8 *
Mbanga	1.11	81.6	90.3	1.15	59.5	68.6	−22.1 *	−21.7 *
Melong	0.97	97.4	94.9	1.10	79.9	88.0	−17.5 *	−6.9 *
Ndom	1.07	44.6	47.5	1.39	34	47.4	−10.6 *	−0.1 *
New Bell	0.97	64.5	62.3	0.99	83.8	83.0	19.3	20.7
Ngambe	1.09	48.7	53.2	0.91	60	54.6	11.3	1.4
Njombe Penja	1.42	80	113.4	1.13	94.2	106.1	14.2	−7.3 *
Nkondjock	0.99	106.4	105.6	0.95	101.8	96.3	−4.6 *	−9.3 *
Nkongsamba	0.99	95.8	94.7	1.07	90.7	97.1	−5.1 *	2.4
Nylon	0.91	90.1	82.4	0.93	94.1	87.8	4	5.4
Pouma	1.09	97.7	106.7	1.46	72.3	105.3	−25.4 *	−1.5 *
Yabassi	1.27	77.9	99.1	1.32	65.6	86.8	−12.3 *	−12.4 *

* The negative sign (−) shows a drop in coverage after the pandemic compared to before, while a positive sign (+) indicates an increase in coverage.

**Table 3:** DTP-1 coverage variations in the pre-pandemic and pandemic period in the Centre Region.

District	2019 Weight	2019 Coverage	Adjusted 2019 Coverage	2020 Weight	2020 Coverage	Adjusted 2020 Coverage	Variation *	Adjusted Variation *
**Akonolinga**	0.94	97.8	92.2	0.93	81.1	75.8	−16.7 *	−16.4 *
**Awae**	0.94	100.9	94.8	0.94	67.3	63.3	−33.6 *	−31.6 *
**Ayos**	0.95	88.2	83.5	1.05	69.9	73.4	−18.3 *	−10.1 *
**Bafia**	0.94	95.3	89.3	0.95	87.5	82.9	−7.8 *	−6.4 *
**Biyem Assi**	0.97	164.2	158.6	0.94	157.7	148.4	−6.5 *	−10.2 *
**Cite Verte**	1.04	89.1	92.3	1.34	82	110.0	−7.1 *	17.6
**Djoungolo**	0.89	93.4	83.4	0.92	86.3	79.3	−7.1 *	−4.1 *
**Ebebda**	0.80	99.7	79.4	0.81	79.8	64.3	−19.9 *	−15.1 *
**Efoulan**	0.96	86	82.6	1.04	84.3	87.9	−1.7 *	5.4
**Elig Mfomo**	0.94	102.5	96.4	0.94	99	93.1	−3.5 *	−3.3 *
**Eseka**	0.91	82.7	75.0	0.91	59.2	53.7	−23.5 *	−21.3 *
**Esse**	0.88	100.7	88.4	0.90	83.8	75.6	−16.9 *	−12.8 *
**Evodoula**	0.94	112.2	105.5	0.95	75.1	71.2	−37.1 *	−34.3 *
**Mbalmayo**	0.98	82.5	81.0	0.93	87.6	81.7	5.1	0.7
**Mbandjock**	1.00	111.9	111.9	0.95	105.3	99.6	−6.6 *	−12.3 *
**Mbankomo**	0.88	131.1	115.4	1.06	107.3	114.1	−23.8 *	−1.3 *
**Mfou**	0.97	128.8	124.3	1.17	142.2	166.5	13.4	42.2
**Monatele**	0.94	81.3	76.4	1.03	72.2	74.3	−9.1 *	−2.1 *
**Mvog-Ada**	0.97	160	154.9	1.01	151.1	152.4	−8.9 *	−2.5 *
**Nanga Eboko**	0.94	89.8	84.4	0.97	68	65.9	−21.8 *	−18.5 *
**Ndikinimeki**	0.95	75.1	71.0	0.94	68.7	64.6	−6.4 *	−6.4 *
**Ngog Mapubi**	0.94	85.5	80.1	0.94	91.8	86.3	6.3	6.2
**Ngoumou**	0.94	96.5	90.7	0.94	86	80.8	−10.5 *	−9.9 *
**Nkolbisson**	0.92	81.8	75.1	0.92	92.4	85.2	10.6	10.1
**Nkolndongo**	0.92	88.7	81.4	0.94	82.6	77.6	−6.1 *	−3.8 *
**Ntui**	0.94	97.5	91.2	0.95	84.9	80.4	−12.6 *	−10.8 *
**Obala**	0.94	105.7	99.7	0.98	76.8	75.4	−28.9 *	−24.2 *
**Odza**	0.94	85.9	80.3	0.93	88	82.0	2.1	1.6
**Okola**	0.96	125.4	120.2	1.07	96.7	103.4	−28.7 *	−16.7 *
**Sa’a**	0.95	95.6	90.5	0.94	91.5	85.8	−4.1 *	−4.7 *
**Soa**	0.94	124.4	116.9	0.97	154.6	149.8	30.2	32.9
**Yoko**	1.03	83.2	85.3	0.94	69	64.9	−14.2 *	−20.4 *

* The negative sign (−) shows a drop in coverage after the pandemic compared to before, while a positive sign (+) indicates an increase in coverage.

**Table 4:** DTP-3 coverage variations in the pre-pandemic and pandemic period in the Centre Region.

District	2019 Weight	2019 Coverage	Adjusted 2019 Coverage	2020 Weight	2020 Coverage	Adjusted 2020 coverage	Variation *	Adjusted Variation *
**Akonolinga**	0.94	99.8	94.1	0.93	83.7	78.2	−16.1 *	−15.9 *
**Awae**	0.94	94.2	88.5	0.94	47.7	44.8	−46.5 *	−43.7 *
**Ayos**	0.95	67.9	64.3	1.05	53.3	56.0	−14.6 *	−8.3 *
**Bafia**	0.94	88.3	82.8	0.95	77.6	73.5	−10.7 *	−9.2 *
**Biyem Assi**	0.97	161.6	156.1	0.94	151.5	142.6	−10.1 *	−13.6 *
**Cite Verte**	1.04	82.6	85.6	1.34	72.5	97.2	−10.1 *	11.6
**Djoungolo**	0.89	87.9	78.5	0.92	79.7	73.2	−8.2 *	−5.2 *
**Ebebda**	0.80	89	70.8	0.81	75.1	60.5	−13.9 *	−10.3 *
**Efoulan**	0.96	89.6	86.0	1.04	85.5	89.2	−4.1 *	3.2
**Elig Mfomo**	0.94	91.3	85.8	0.94	82.7	77.7	−8.6 *	−8.1 *
**Eseka**	0.91	84.7	76.8	0.91	55.9	50.7	−28.8 *	−26.1 *
**Esse**	0.88	90.9	79.8	0.90	71.7	64.7	−19.2 *	−15.1 *
**Evodoula**	0.94	98	92.1	0.95	76.5	72.5	−21.5 *	−19.6 *
**Mbalmayo**	0.98	71.4	70.1	0.93	76.3	71.2	4.9	1.0
**Mbandjock**	1.00	85.1	85.1	0.95	86.9	82.2	1.8	−2.9 *
**Mbankomo**	0.88	115.6	101.7	1.06	89.2	94.9	−26.4 *	−6.9 *
**Mfou**	0.97	118	113.9	1.17	126.3	147.8	8.3	34.0
**Monatele**	0.94	75.5	71.0	1.03	61.4	63.2	−14.1 *	−7.8 *
**Mvog-Ada**	0.97	155.5	150.5	1.01	139.7	140.9	−15.8 *	−9.6 *
**Nanga Eboko**	0.94	73.4	69.0	0.97	57.6	55.8	−15.8 *	−13.2 *
**Ndikinimeki**	0.95	73.1	69.1	0.94	64.7	60.8	−8.4 *	−8.3 *
**Ngog Mapubi**	0.94	83.2	78.0	0.94	84.5	79.4	1.3	1.5
**Ngoumou**	0.94	92.3	86.8	0.94	79.2	74.4	−13.1 *	−12.3 *
**Nkolbisson**	0.92	76.9	70.6	0.92	86.1	79.3	9.2	8.8
**Nkolndongo**	0.92	80.7	74.1	0.94	72.7	68.3	−8 *	−5.7 *
**Ntui**	0.94	84.5	79.0	0.95	67.7	64.1	−16.8 *	−14.9 *
**Obala**	0.94	101.8	96.0	0.98	71	69.7	−30.8 *	−26.2 *
**Odza**	0.94	85	79.5	0.93	82.7	77.0	−2.3 *	−2.5 *
**Okola**	0.96	113.6	108.9	1.07	88.7	94.9	−24.9 *	−14.0 *
**Sa’a**	0.95	87.8	83.1	0.94	84.4	79.1	−3.4 *	−4.0 *
**Soa**	0.94	125.3	117.8	0.97	146.2	141.7	20.9	23.9 *
**Yoko**	1.03	65.5	67.1	0.94	47.3	44.5	−18.2 *	−22.7 *

* The negative sign (−) shows a drop in coverage after the pandemic compared to before, while a positive sign (+) indicates an increase in coverage.

## Data Availability

Data from the corresponding author of this research is available upon reasonable request. However, the Cameroon DHIS-2 databased was used as the data source.
